# Highly Absorbent Antibacterial Hemostatic Dressing for Healing Severe Hemorrhagic Wounds

**DOI:** 10.3390/ma9090793

**Published:** 2016-09-21

**Authors:** Ting-Ting Li, Ching-Wen Lou, An-Pang Chen, Mong-Chuan Lee, Tsing-Fen Ho, Yueh-Sheng Chen, Jia-Horng Lin

**Affiliations:** 1 Innovation Platform of Intelligent and Energy-Saving Textiles, School of Textiles, Tianjin Polytechnic University, Tianjin 300387, China; tingtingli@tjpu.edu.cn; 2 Department of Chemistry and Chemical Engineering, Minjiang University, Fuzhou 350108, China; cwlou@ctust.edu.tw; 3 Graduate Institute of Biotechnology and Biomedical Engineering, Central Taiwan University of Science and Technology, Taichung 40601, Taiwan; mclee@ctust.edu.tw; 4 Laboratory of Fiber Application and Manufacturing, Department of Fiber and Composite Materials, Feng Chia University, Taichung 40724, Taiwan; duncannano@gmail.com; 5 Department of Medical Laboratory Science and Biotechnology, Central Taiwan University of Science and Technology, Taichung 40601, Taiwan; tfho@ctust.edu.tw; 6 Department of Biomedical Imaging and Radiological Science, China Medical University, Taichung 40402, Taiwan; yuehsc@mail.cmu.edu.tw; 7 School of Chinese Medicine, China Medical University, Taichung 40402, Taiwan; 8 Department of Fashion Design, Asia University, Taichung 41354, Taiwan

**Keywords:** nonwoven, fiber technology, nanosilver, hemostatic, wound dressing

## Abstract

To accelerate healing of severe hemorrhagic wounds, a novel highly absorbent hemostatic dressing composed of a Tencel^®^/absorbent-cotton/polylactic acid nonwoven base and chitosan/nanosilver antibacterial agent was fabricated by using a nonwoven processing technique and a freeze-drying technique. This study is the first to investigate the wicking and water-absorbing properties of a nonwoven base by measuring the vertical wicking height and water absorption ratio. Moreover, blood agglutination and hemostatic second tests were conducted to evaluate the hemostatic performance of the resultant wound dressing. The blending ratio of fibers, areal weight, punching density, and fiber orientation, all significantly influenced the vertical moisture wicking property. However, only the first two parameters markedly affected the water absorption ratio. After the nonwoven base absorbed blood, scanning electron microscope (SEM) observation showed that erythrocytes were trapped between the fibrin/clot network and nonwoven fibers when coagulation pathways were activated. Prothrombin time (PT) and activated partial thromboplastin time (APTT) blood agglutination of the resultant dressing decreased to 14.34 and 50.94 s, respectively. In the femoral artery of the rate bleeding model, hemostatic time was saved by 87.2% compared with that of cotton cloth. Therefore, the resultant antibacterial wound dressing demonstrated greater water and blood absorption, as well as hemostatic performance, than the commercially available cotton cloth, especially for healing severe hemorrhagic wounds.

## 1. Introduction

Personalized medicine is increasingly becoming mainstream with the aging society and low birth rate for humans, with particular attention given to perfect medical care. However, some circumstances such as traffic accidents, wartime firearms, typhoons, earthquakes, and tsunamis occasionally occur by surprise. These disasters may result in massive bleeding, which can trigger coma, shock, and even death for injured humans. During surgical operation, reducing the bleeding time and shortening the operation duration can significantly influence a patient’s prognosis.

Wound dressing composed of collagen, chitosan, and sodium alginate [1,2,3] has extensive applications. Arafat et al. and Tronci et al., conducted many related studies on collagen and collagen wound dressing; in which they demonstrated that these dressings can achieve a certain mechanical strength, high swelling ratio, and triple helical features [4,5,6].

Blood comprises a large amount of water. Thus, hemostatic materials should be highly absorbent. As a cellulose fiber, Tencel^®^ fiber contains a high proportion of swelling fibril bundles and presents moisture wicking properties [7]. The fiber structure and water-absorbing degree can determine the good skin affinity of Tencel^®^ fiber. Moreover, this fiber demonstrates excellent moisture management capacity and absorbs water molecules into fiber molecules; thus, no excessive water remains on the superficial skin, and bacteria cannot grow, avoiding discomfort in people [8]. Polylactic acid (PLA) fiber is a biodegradable material with excellent biocompatibility; therefore, PLA fiber is widely used in drug controlled release, tissue engineering, and wound dressing. PLA fiber contains numerous carboxylate groups and presents a relatively hydrophobic character and brittle strength [9]. Absorbent cotton (AC) demonstrates excellent moisture-absorbing and water-retaining properties, and absorbed molecules can be successfully trapped among AC fibers without producing gravity water. However, fine fibers are prone to remain in the wounds even after removal [10]. Perfect wound dressing should exhibit (i) moisture-retaining properties; (ii) avoidance of secondary infection; (iii) blood and interstitial fluid absorption; (iv) excellent coverage and tear resistance; (v) good biocompatibility; and (vi) rapid healing [11,12,13,14]. Therefore, the blending of these fibers aims to provide appropriate strength, good biocompatibility, good moisture-retaining properties, and rapid blood absorption for resultant nonwoven dressings. Furthermore, such blending leads to quick hemostasis and creates a fast-healing moist environment for wound prognosis.

For healing severe hemorrhagic wounds, conventional hemostatic methods include digital pressure hemostasis, pressure dressing hemostasis, and tourniquet hemostasis. In particular, pressure dressing hemostasis results in successful hemostasis but it easily triggers complications if an inexperienced person performs the operation. Therefore, as a simple hemostatic method, a novel multi-component and porous nonwoven base composited with chitosan and silver nanoparticles can be directly pressed on the severe hemorrhagic spot to induce hemostasis.

During healing, microbial infection leads to inflammation [15]. Therefore, the antibacterial property becomes a required alternative for wound dressing. Chitosan, as a biodegradable, nontoxic, complex carbohydrate derivative of chitin demonstrates mucoadhesive activity and it can be considered an ideal candidate as a hemostatic agent [16]. This compound carries positive ions in acidic condition because of the amido group and combines with electronegative bacterial cells; the osmotic pressure between inside and outside of bacterial cells is then altered, resulting in antibacterial property. Moreover, chitosan offers excellent wound-healing potential [17], but such potential is limited to minor hemorrhages and insufficient to assess the potential efficacy of severe bleeding.

Nanosilver particles were reported to exert more significant antibacterial effects; therefore, the synthesis, characterization, and antibacterial activities of Ag nanoparticles in various approaches have become major topics for research [18,19,20,21,22,23,24,25,26,27,28,29,30,31,32,33,34]. Among these processes, the conventional synthesis of nanosilver particles uses large amounts of harmful solvents and reagents, such as hydrazine (N_2_H_4_), sodium borohydride (NaBH_4_), sodium hypophosphite (NaH_2_PO_2_), and formaldehyde (HCHO). Based on a green sustainable chemistry concept that aims to reduce the amounts of raw materials harmful to the human body and environment, as well as decrease the usage of catalysts and solvents and the generation of unnecessary chemical derivatives and their associated wastes, a novel green synthesis method was proposed to synthesize Ag nanoparticles in our laboratory by using chitosan and silver nitrate solution. The chitosan-encapsulated Ag nanoparticles were confirmed to be effective antibacterial agents against *Escherichia coli* and *Staphylococcus aureus* [35,36].

When fabricating a highly absorbent wound dressing, a highly absorbent structure and excellent blood-retaining property are essential. Although PLA fibers are relatively hydrophobic polymers that limit water uptake, these fibers can provide a supporting efficiency for nonwoven structures and absorb large amounts of blood in the nonwoven base. The blood is then held in the structure to prevent the blood from entering into the nonwoven base, thereby avoiding the production of gravity water and ejection of the blood. Consequently, a multi-component nonwoven structure containing Tencel^®^ fibers, highly absorbent cotton, and PLA fibers was designed as a wound dressing base. Moreover, antibacterial chitosan-encapsulated Ag nanoparticles were doped into the nonwoven base. This study focused on the nonwoven composition and process parameters influencing the blood absorption-related properties, wicking and water-absorbing capabilities, and hemostatic performance in severe hemorrhage of rat femoral artery.

## 2. Experimental Section

### 2.1. Materials and Methods

In this experiment, 1.7 D Tencel^®^ fiber (Taiwan Web-Pro Co., Ltd., Kaohsiung, Taiwan), 1.6 D PLA fiber (Far Eastern New Century Corporation, Taipei, Taiwan), and 1.53 D absorbent-cotton (AC) fiber (Asiatic Fiber Corporation, Taipei, Taiwan) were fabricated into a Tencel^®^/AC/PLA (TCP) nonwoven structure via processes including opening, blending, carding, lapping, and needle punching. The ratios of Tencel^®^ fiber and PLA fiber in the nonwoven base were set as 40:40, 50:30, 60:20, 70:10, and 80:0 wt %/wt %, and the AC fiber was set as 20 wt %. The punching density was set as 50, 75, and 100 punches/cm^2^, and the areal weight was set as 100, 150, and 200 g/m^2^. The effects of processing parameters on the vertical wicking height and water absorption ratio of TCP composite nonwoven structures were investigated. Blood absorption of nonwoven structures was also compared. The name codes of fabricated samples used for blood absorption are displayed in Table 1.

**Figure 1:**
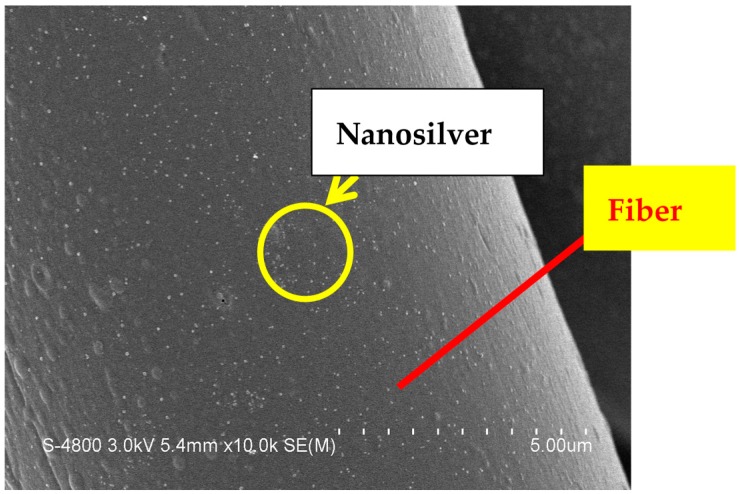
Scaning electron microscopy (SEM) observation of nanosilver-doped nonwoven fibers.

About 80% of chitosan powder (Global Biological Technology Co., Ltd., Tainan, Taiwan) was dissolved in acetic acid by stirring at 25 ± 2 °C. Pure-grade silver nitrate solution (purchased from Union Chemical Works Ltd., Hsinchu, Taiwan) was then blended with the aforementioned chitosan solution to create a chitosan/nanosilver antibacterial agent. The green synthesis of the antibacterial agent was performed as described in our previous study [35]. The antibacterial agent was coated on the TCP nonwoven by free-drying, forming the resultant wound dressing. As a result, nanosilver was successfully covered on the surface of fibers (Figure 1).

### 2.2. Measurements

#### 2.2.1. Vertical Wicking Height

Five pieces of nonwovens along the cross machine direction (CD) and machine direction (MD) were cut into 200 mm (length) × 25 mm (width) in accordance with the standard of CNS 5611. Each piece was vertically fastened on a horizontal clamper placed on top of a water tank. After soaking each piece in water for 10 min, the vertical wicking height of each piece with different fiber blendings was determined.

#### 2.2.2. Water and Blood Absorption

Each group of TCP nonwovens was cut into 50 mm × 50 mm pieces. The weight of each piece was recorded as W_0_. Each piece was then placed into a 250 mL beaker containing 80 mL of water and blood. Fresh and healthy whole blood was acquired from volunteers. After soaking the pieces in water and blood for 5 min, each piece was weighed as *W_wet_*. The water absorption ratio (*W*) was calculated as follows:

*W = (W_wet_* − *W*_0_*)/W*_0_ (1)

#### 2.2.3. Mechanical Property

The tensile strength along the MD and CD of TCP nonwovens was tested in accordance with the standard of ASTM D5035-11. The Instron 5566 universal testing machine (Instron, Norwood, MA, USA) was used to measure the rectangular specimens. The crosshead speed was 300 mm/min. The tensile specimen measured 180 mm long and 25.4 mm wide.

Burst strength of TCP nonwovens was determined using Instron 5566 Tester (Instron, Norwood, MA, USA) in accordance with the standard of ASTM D1883. The semicircular-ended head (25 mm in diameter) attached to a 10 kN load cell was driven onto the nonwovens at a speed of 100 mm/min.

#### 2.2.4. PT and APTT Experiments

The blood test in this work was approved by the Institutional Animal Care and Use Committee (IACUC) with Approval No. 100-CTUST-22 and a protocol period going from 1 June 2012 to 5 May 2015. Prothrombin time (PT) and activated partial thromboplastin time (APTT) were measured to determine the effect of wound dressing on blood-clotting time. The PT test was performed on heparinized plasma, and the PT reagent comprised tissue factor, CaCl_2_, and phospholipid. The APTT test was performed by adding APTT reagent into heparinized plasma. The APTT reagent was composed of plasma activator, phospholipid, and CaCl_2_. The PT agent (APTT reagent) and wound dressing sample were added to plasma in the test tube, and PT (APTT) was tested at 37 °C simultaneously.

#### 2.2.5. Animal Experiment

Femoral artery bleeding of Wistar rats (purchased from BioLASCO Taiwan Co., Ltd., Taipei, Taiwan) was performed to evaluate hemostasis. A hemostatic wound dressing was then pressed on the bleeding site lightly for 2 s to absorb excessive blood voluntarily until hemostasis. The hemostasis time was expressed as the time between artery excision and bleeding stop.

#### 2.2.6. Statistical Analysis

Statistical analysis of vertical wicking and water absorption, as well as hemostatic performance, was performed using one-way ANOVA with SPSS software (IBM, Chicago, IL, USA). Differences were considered statistically significant at * *p* < 0.05 and ** *p* < 0.01.

## 3. Results and Discussion

### 3.1. Wicking and Water Absorption of Nonwoven Wound Dressing

**Figure 2:**
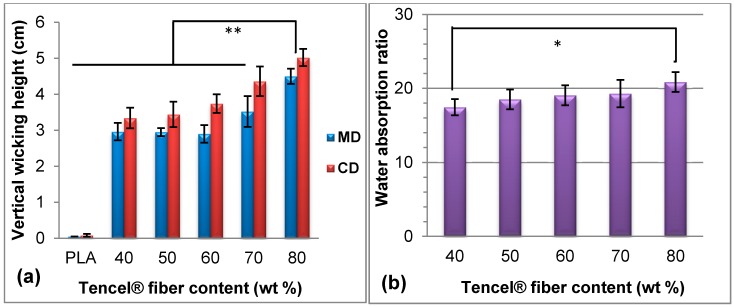
(**a**) Vertical wicking height and (**b**) water absorption of Tencel^®^/Absorbent-Cotton/PLA (TCP) nonwovens with various fiber blendings. ** *p* < 0.01; * *p* < 0.05.

Figure 2 shows the vertical wicking and water absorption properties of TCP nonwovens with different Tencel^®^ fiber contents. The PLA fiber, which was composed of a thermoplastic aliphatic polyester polymerized by lactide and lactic acid, demonstrated weak water absorption. This finding was ascribed to the byproducts formed in the carboxyl polymerization reaction, which did not occur in hydrophilic groups. In addition, given that PLA fibers exhibit high crystallinity, water molecules could not easily enter into the amorphous region. As a result, the Tencel^®^ fiber contents significantly affected the vertical wicking weight of water molecules. Figure 2a shows that the vertical wicking height increased with the blending ratio of Tencel^®^ fibers, because a higher number of molecular chains of Tencel^®^ fibers contained more hydrophilic groups (e.g., OH groups), which could easily produce chemical-bonded water. This finding explained the vertical wicking height tendency of TCP nonwovens, similar to the water absorption illustrated in Figure 2b, in which the water absorption ratio steadily increased with the Tencel^®^ fiber content. Figure 2a shows that CD displayed significantly higher wicking height than MD because the nonwoven base along CD contained a higher fiber content and more water molecules, capillaries, and channels [37]. About 80 wt % Tencel^®^ fibers in nonwovens revealed very significantly higher vertical wicking height (** *p* < 0.01) and significant water absorption ratio (* *p* < 0.05) than 40 wt % Tencel^®^ fibers in nonwovens.

**Figure 3:**
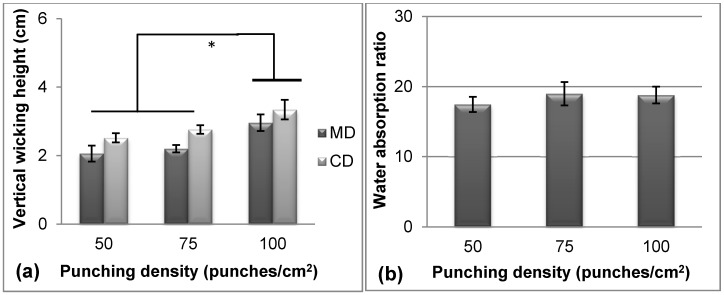
(**a**) Vertical wicking height and (**b**) water absorption of TCP nonwovens with various punching densities. * *p* < 0.05.

Figure 3 shows the effect of punching density on vertical wicking height and water absorption ratio. In particular, the Tencel^®^ fiber content was 40 wt %, and the areal weight was 100 g/m^2^. Figure 3a shows that the vertical wicking height increased with punching density from 50 punches/cm^2^ to 100 punches/cm^2^ because the contacting area between adjacent fibers increased with punching density. Circuitous water-absorbing channels also increased in number. This finding agreed well with the results of Çil et al., who reported that compact fabrics exhibit enhanced vertical wicking properties because of the good capillarity effect [38]. However, Figure 3b shows that the punching density was insignificantly correlated with the water absorption ratio. This finding demonstrated that fiber entanglements and nonwoven thickness reached a saturation state at 50–100 punches/cm^2^ and could not promote moisture holding capacity among porous structures after thickness compression [39,40].

**Figure 4:**
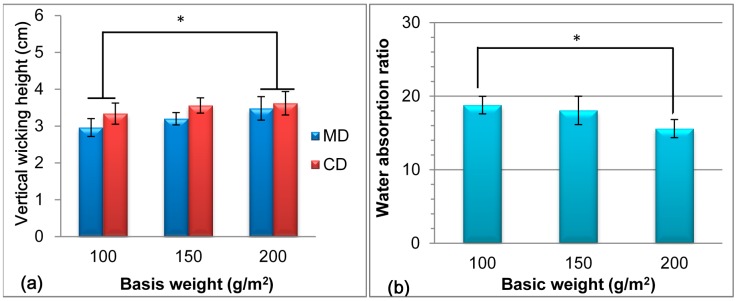
(**a**) Vertical wicking height and (**b**) water absorption of TCP nonwovens with various basal weights. * *p* < 0.05.

Figure 4 shows the vertical wicking height and water absorption ratio of nonwovens with various areal weights, which significantly influenced the wicking and water-absorbing properties. As shown in Figure 4a, the vertical wicking height steadily improved with the areal weight of the nonwoven. The nonwoven exhibited a porous structure, and liquid penetrated through the pores at extra pressure and capillary effect. With increasing areal weight of the nonwoven, the fiber content per unit volume increased, which led to frequent water diffusion and strong capillarity in each direction. A rapid diffusion rate and improved wicking property were both achieved when water diffused merely along the surface, as indicated by Duru et al. and Laughlin et al. [41,42]. Figure 4b shows that the water absorption ratio decreased progressively as the areal weight increased from 100 g/m^2^ to 200 g/m^2^. The nonwoven exhibited a porous structure, and its fiber swelled after water absorption. However, a high areal weight of nonwoven indicated a high fiber content, and high additional compression from contacts of water molecules generated gravity water in a given space, so the water molecules presented difficulty in holding in the porous structure.

The different trends and significance of the vertical wicking height and water absorption ratio with the Tencel^®^ content, punching density, and areal weight (Figure 3 and Figure 4) were attributed to different mechanisms. The vertical wicking height was attributed to the water diffusion rate, and water absorption resulted from porosity and structure. Therefore, CD nonwoven demonstrated high fiber content and rapid diffusion rate, which resulted in high vertical wicking height, as displayed in Figure 2a, Figure 3a, and Figure 4a. When the nonwoven contained 80 wt % Tencel^®^ fibers, a weight of 100 g/m^2^, and a punching density of 100 punches/cm^2^, the vertical wicking height and water absorption ratio along CD reached 5.0 cm and 20.9 times, respectively.

### 3.2. Mechanical Properties of Nonwoven Wound Dressings

**Figure 5:**
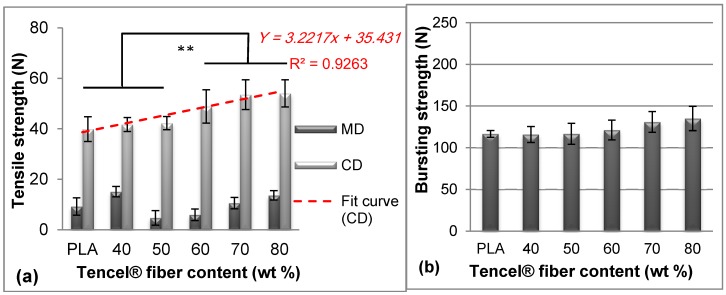
(**a**) Tensile strength and (**b**) burst strength of TCP nonwovens with various Tencel^®^ fiber contents, punching density of 100 punches/cm^2^, and areal weight of 100 g/m^2^. ** *p* < 0.01.

When absorbing a large amount of interstitial fluid, the fiber structure becomes loose and has a relatively low mechanical property, which brings inconvenience to medical workers. Moreover, the burst property can simulate the buckling load of wound dressing when applied on wrist, elbow, knee, and heel wounds. Therefore, fiber blendings that influence the mechanical properties are important to support the application of wound dressing. Figure 5 shows the mechanical properties of nonwoven wound dressings with different Tencel^®^ fiber amounts. In Figure 5a, the maximum breaking strength was enhanced when the Tencel^®^ fiber content increased. When Tencel^®^ fiber increased to 60 wt %, the improvement for tensile strength revealed very significant (** *p* < 0.01). This finding demonstrated that Tencel^®^ fiber addition could make up the strength of the whole nonwoven dressing compared with pure PLA nonwoven, because the Tencel^®^ fiber presented a tenacity of 3.2 g/denier higher than that of the PLA fibers [43]. Additionally, the tensile strength (*Y*) displayed a highly linear correlation with the Tencel^®^ fiber content (*x*), as expressed in Equation (2).

Y = 3.2217x + 35.431
(2)

When the Tencel^®^ addition increased to 60%, its improvement to tensile strength became very significant. However, the burst strength was insignificantly related to the Tencel^®^ fiber content, as shown in Figure 5b. At the same areal weight, the fiber web in the thickness direction exhibited a relatively higher fiber volume density, even with different Tencel^®^ fibers, and the compactness and entanglement interactively affected tensile load shared by the individual fibers [44]. Therefore, the burst strength did not significantly increase with the Tencel^®^ fiber content, in contrast to the tensile strength results in Figure 5a. In conclusion, the nonwoven dressing base presented a tensile stress of 1.9–2.5 MPa and burst strength of 116–135 N, which could reach the mechanical levels of the wound dressing application.

### 3.3. Blood Absorption of Nonwoven Wound Dressing

**Figure 6:**
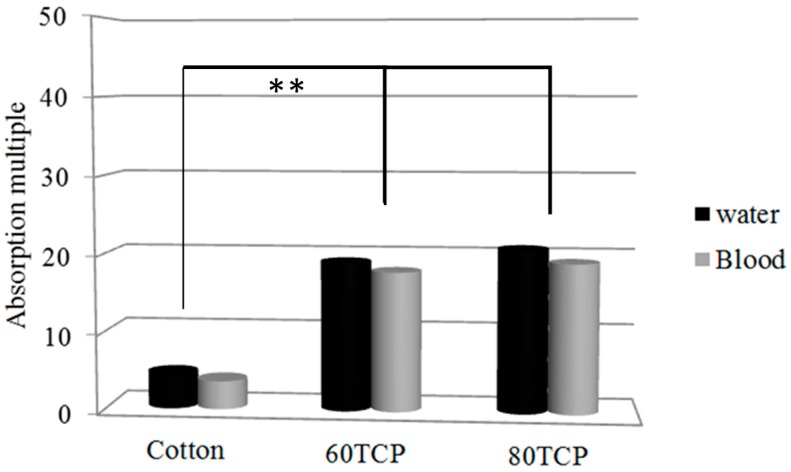
Water and blood absorptions of cotton cloth and TCP nonwovens with 60 wt % and 80 wt % Tencel^®^ fibers. ** *p* < 0.01.

Figure 6 shows that blood absorption with increasing Tencel^®^ fiber content was similar to water absorption because blood consists of 55% plasma, which contains 92% water. Cotton cloth absorbed 4.5 times water and 3.6 times blood compared with its dry weight. Comparatively, water and blood absorptions reached 21.1 and 19.3 times for 80 TCP and 19.7 and 18.6 times for 60 TCP. Therefore, water and blood absorptions of TCP nonwoven were four to five times that of cotton cloth. The improvement in water and blood absorptions compared with cotton cloth was very significant (** *p* < 0.01), indicating the superior blood absorption capacity for the TCP nonwoven base. The blood absorption efficiency of the composite nonwoven reached 38 g/m^2^, which was equivalent to that of various fabric dressings, ranging from 20 g/m^2^ to 42 g/m^2^, as reported by Terrill et al. [45].

**Figure 7:**
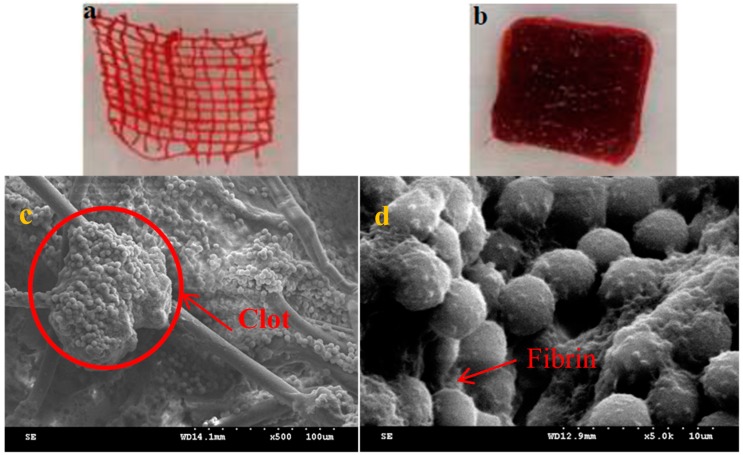
(**a**) Cotton cloth after blood absorption; (**b**) TCP nonwoven after blood absorption. SEM observation of TCP nonwoven after blood absorption (**c**: 500×; **d**: 5000×).

Figure 7 shows the observations of cotton cloth and TCP nonwoven after blood absorption. A significant difference was found in blood absorption for cotton cloth and TCP nonwoven, upon comparing Figure 7a,b. Blood was absorbed only in fibers for cotton cloth, but it filled between the interspace of nonwoven fibers besides the fibrous part. This finding suggested the superior blood and water absorption for the TCP nonwoven base. Figure 7c,d show that numerous erythrocytes (with diameters of 4–6 μm) aggregated between fibers, and the coagulation reaction still occurred and formed clots. Although erythrocytes were in contact with nonwoven fibers, they were still trapped between the fibrin network and attached to the surface of nonwoven fibers when coagulation pathways were activated, which is explained by the cellular morphology of fibrin around the erythrocytes [46]. Therefore, the nonwoven fibers used in this study could not influence the coagulation mechanism but may be applied for future resultant hemostatic wound dressing.

### 3.4. Hemostatic Performance of Nonwoven Wound Dressing

**Figure 8:**
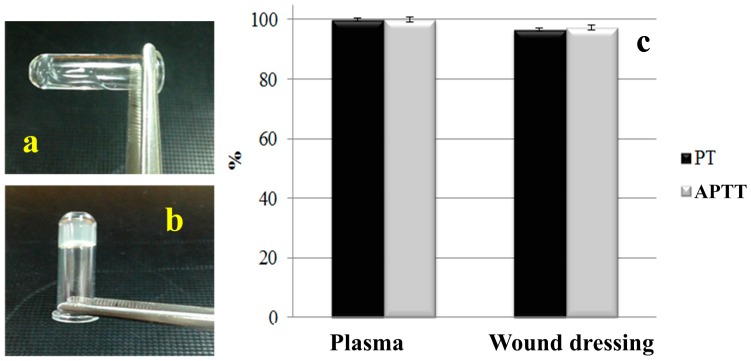
(**a**) Plasma; (**b**) its agglutination reaction and (**c**) prothrombin time (PT) and activated partial thromboplastin time (APTT) of wound dressing.

Figure 8 shows the PT and APTT of plasma and TCP nonwoven. Plasma was directly separated from the blood. Blood is originally a fluid, flowing along the horizontal test tube (Figure 8a). After activation, the agglutination reaction occurred, resulting in blood coagulation (Figure 8b). To prolong PT and APTT, heparin was added into plasma. Figure 8 displays that the clotting times of plasma in PT and APTT were 14.85 and 52.3 s, respectively, and those with antibacterial agent (resultant wound dressing) were 14.34 (PT) and 50.94 s (APTT). Therefore, the PT and APTT of wound dressing decreased to 96.6% and 97.4%, compared with those of plasma. The shortened time was due to platelets in plasma, which came into contact with chitosan in the antibacterial agent to generate an activated reaction, accompanied with extended and deformed pseudopodia, activated integrin compounds, calcium ion signal starting with platelet adherence, and accelerated combination fibrin monomer [47]. However, the PT and APTT of the resulting samples surpassed that of the control group (plasma) within less than 3 s, demonstrating that wound dressing decreased the clotting time in PT and APTT insignificantly [48].

**Figure 9:**
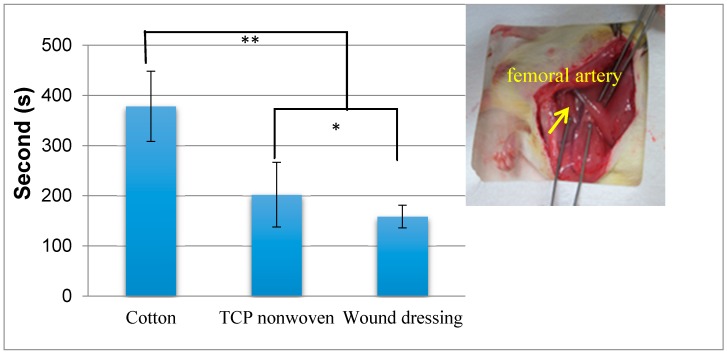
Comparisons of the hemostasis time of rat femoral artery injury by using cotton cloth (cotton), TCP nonwoven (TCP), and resultant wound dressing. ** *p* < 0.01; * *p* < 0.05.

Figure 9 shows the hemostasis time of rat femoral artery injury with various hemostatic materials. The hemostasis time of cotton cloth was 380 s, and that of TCP nonwoven was shortened to 180 s, a 52.6% decrease. The significant reduction in the hemostasis time for TCP nonwoven occurred because the TCP nonwoven, with a highly absorbent character, absorbed a large amount of blood quickly and facilitated the platelet agglutination reaction on the wound. The hemostasis time of wound dressing was 157 s, which significantly decreased by 45 s compared with that of the TCP nonwoven base (* *p* < 0.05). This finding indicates that the addition of antibacterial agent improved the hemostatic performance because the chitosan carrying positive ions triggered agglutination of erythrocytes and blood platelets, and plasma proteins were absorbed on the surface of the wound dressing upon contact with blood [36]. Nanosilver addition may reduce the bleeding amount and shorten the bleeding time, thereby demonstrating a hemostasis effect [35]. One-way ANOVA results indicated that the hemostasis time of wound dressing was also significantly shortened by 220 s compared with that of cotton cloth (** *p* < 0.01).

## 4. Conclusions

In this work, we successfully fabricated a highly absorbent antibacterial hemostatic wound dressing composed of TCP nonwoven and then impregnated with chitosan/nanosilver antibacterial agent. The effects of nonwoven processing parameters on the vertical wicking property and the water and blood absorption of the TCP nonwoven were evaluated. Results showed that the vertical wicking height significantly increased with the Tencel^®^ fiber content, punching density, and basis weight. Evidently, the water absorption ratio was correlated with the Tencel^®^ fiber content and areal (basis) weight. Besides, the tensile strength of the TCP nonwoven increased significantly in relation to the Tencel^®^ fiber content. The resulting nonwoven dressing base presented a tensile stress of 1.9–2.5 MPa and burst strength of 116–135 N, which could reach the mechanical levels of the wound dressing application. The TCP nonwoven showed very significant improvement in blood absorption compared with cotton cloth. Scaning electron microscopy (SEM) observations revealed that erythrocytes were in contact with nonwoven fibers, but they were successfully entrapped in the fibrin network and attached on the surface of nonwoven fibers. The PT and APTT time and hemostatic time in the rat femoral artery model of the wound dressing were further explored. Overall, the results indicated that the clotting time of wound dressing in PT and APTT array decreased to 14.34 and 50.94 s, respectively. Remarkably, the addition of antibacterial agent shortened the hemostatic time by 45 s. These findings showed that the wound dressing in this study is a viable option for hemostatic wound dressing, especially in severe bleeding injuries.

## Figures and Tables

**Table 1:** Name codes of Tencel^®^/Absorbent-Cotton/PLA (TCP) composite nonwoven used for blood absorption.

Composite Nonwoven	Tencel^®^ (wt %)	AC (wt %)	PLA (wt %)	Punching Density (Punches/cm^2^)	Areal Weight (g/m^2^)	Thickness (mm)
40TCP	40	20	40	100	100	0.85
50TCP	50	20	30	100	100	0.85
60TCP	60	20	20	100	100	0.85
70TCP	70	20	10	100	100	0.85
80TCP	80	20	0	100	100	0.85
